# Sport and Transgender People: A Systematic Review of the Literature Relating to Sport Participation and Competitive Sport Policies

**DOI:** 10.1007/s40279-016-0621-y

**Published:** 2016-10-03

**Authors:** Bethany Alice Jones, Jon Arcelus, Walter Pierre Bouman, Emma Haycraft

**Affiliations:** 1 Nottingham Centre for Gender Dysphoria, 3 Oxford Street, Nottingham, NG1 5BH UK; 2 School of Sport, Exercise, and Health Sciences, Loughborough University, Loughborough, UK; 3 Division of Psychiatry and Applied Psychology, Faculty of Medicine and Health Sciences, University of Nottingham, Nottingham, UK

**Keywords:** Gender Identity, Sport Participation, Competitive Sport, Sport Organisation, International Olympic Committee

## Abstract

**Background:**

Whether transgender people should be able to compete in sport in accordance with their gender identity is a widely contested question within the literature and among sport organisations, fellow competitors and spectators. Owing to concerns surrounding transgender people (especially transgender female individuals) having an athletic advantage, several sport organisations place restrictions on transgender competitors (e.g. must have undergone gender-confirming surgery). In addition, some transgender people who engage in sport, both competitively and for leisure, report discrimination and victimisation.

**Objective:**

To the authors’ knowledge, there has been no systematic review of the literature pertaining to sport participation or competitive sport policies in transgender people. Therefore, this review aimed to address this gap in the literature.

**Method:**

Eight research articles and 31 sport policies were reviewed.

**Results:**

In relation to sport-related physical activity, this review found the lack of inclusive and comfortable environments to be the primary barrier to participation for transgender people. This review also found transgender people had a mostly negative experience in competitive sports because of the restrictions the sport’s policy placed on them. The majority of transgender competitive sport policies that were reviewed were not evidence based.

**Conclusion:**

Currently, there is no direct or consistent research suggesting transgender female individuals (or male individuals) have an athletic advantage at any stage of their transition (e.g. cross-sex hormones, gender-confirming surgery) and, therefore, competitive sport policies that place restrictions on transgender people need to be considered and potentially revised.

## Key points

**Table:** 

The majority of transgender people have a negative experience when engaging in competitive sports and sport-related physical activity.
There is no direct and consistent research to suggest that transgender female individuals (and transgender male individuals) have an athletic advantage in sport and, therefore, the majority of competitive sport policies are discriminatory against this population.
There are several areas of future research required to significantly improve our knowledge of transgender people’s experiences in sport, inform the development of more inclusive sport policies, and, most importantly, enhance the lives of transgender people, both physically and psychosocially.

## Introduction

Transgender people are those who experience incongruence between the gender that they were assigned at birth (based on the appearance of their genitals) and their gender identity/experienced gender. Gender identity, or experienced gender, can be defined as a person’s internal sense of gender, whether this be male, female, neither or somewhere along the gender continuum. Some transgender people, but not all, will choose to affirm their gender identity by socially transitioning (i.e. living as their experienced gender socially, at work or at an educational institution, with friends and family, outside the home) and some, in addition, will choose to medically transition with cross-sex hormones and gender-confirming surgeries [1, 2]. Although over time various different terms have been used, the term ‘transgender female individual’ will be used to describe individuals assigned male at birth, based on their genital appearance, but who later identify as female. ‘Transgender male individual’ will be used to describe people who are assigned female at birth, based on their genital appearance, but later identify as male. ‘Cisgender’ will be used to describe people who do not experience incongruence between their gender assigned at birth and their gender identity.

Recent reports indicate that the number of transgender individuals who attend transgender health services has increased substantially over the years in many European countries [3–5]. There has also been a significant increase in the number of people who self-identify as transgender and do not necessarily attend transgender health services [6]. For example, Kuyper and Wijsen [6] found that 4.6 % of people who were assigned male at birth and 3.2 % of people who were assigned female at birth in their Dutch population sample reported an ambivalent gender identity (equal identification with the other gender as with the gender they were assigned at birth). The authors also reported that 1.1 % of the people who were assigned male at birth and 0.8 % of the people who were assigned female at birth identified as transgender. It remains unknown how many of these people will seek treatment through a transgender health service. The increase in people who identify as transgender may be at least partly explained by the increase in visibility of transgender people within Western society [4, 5]. For example, Caitlin Jenner, a former athlete and current television personality, recently came out as transgender during a television interview that was viewed all over the world [7]. Increases in visibility may have prompted some people to reflect and question their gender identity [8].

Some transgender people experience stigma, transphobia, prejudice, discrimination and violence as a consequence of their gender identity [9–11]. Ellis et al. [12] found that transgender people were more likely to avoid situations when they were afraid of being harassed, identified as transgender or ‘outed’, such as in clothes shops, public toilets and gyms. Gyms are a popular outlet to engage in sport-related physical activities (i.e. gym fitness exercises) and therefore it is important to create an inclusive environment given the established mental and physical health benefits of physical activity and sport [13, 14]. This is particularly important for transgender people as they have been found to report a high prevalence of depression and anxiety [15, 16], which could be managed with physical activity. Furthermore, physical activity and sport can also contribute towards maintaining the appropriate weight necessary to undergo gender-confirming surgery, acknowledging that not every transgender person will wish to do so [1, 2, 17].

The premise of competitive sport is fairness (i.e. inclusion in the absence of advantage) and, owing to fears surrounding the perceived athletic advantage of transgender people, the question of whether transgender people should be permitted to compete in accordance with their gender identity has been raised and greatly contested within the literature, among sport organisations, fellow competitors and spectators. It is a commonly held belief that androgenic hormones (especially testosterone) confer an athletic advantage in competitive sport. Therefore transgender female individuals, because of high endogenous testosterone levels, are perceived to hold an advantage in sport (when testosterone has not been blocked to a cisgender female level). Transgender men are not thought to possess an athletic advantage, despite being injected with testosterone if they chose to medically transition with cross-sex hormones. However, there has been a paucity of research that has directly explored how androgenic hormone levels are associated with athletic competence in both cisgender and transgender populations (e.g. running time).

To facilitate the inclusion of transgender competitors, in 2004, the International Olympic Committee (IOC) [18] announced that transgender people could participate in all future Olympic games providing they had fully medically transitioned (i.e. had been prescribed cross-sex hormone treatment for 2 years and undergone gender-confirming surgery). Although the requirements of this policy appear to concur with the commonly held belief that transgender people hold an athletic advantage, they have been criticised for not being underpinned by an evidence-based rationale [19]. The IOC [20] has recently updated its policy to be more inclusive of transgender athletes (i.e. fewer restrictions); however, the 2004 policy has been extremely influential on other sport organisations’ policy development. The new (2016) IOC policy will be considered in Sect. 3.

In an attempt to draw a consensus as to whether transgender people should be able to compete in accordance with their gender identity, in 2005 Reeser [21] conducted a review of the literature pertaining to gender identity issues in competitive (elite) sport. Reeser paid particular attention to the evolution of gender verification in competitive sport and whether current competitive sport policies for transgender people are fair. He concluded that, while gender verification has made significant advances, there is a lack of physiological performance-related data in transgender people. This is preventing an overall consensus from being made as to whether transgender sport policies are fair or not (i.e. fairness in the absence of advantage). Reeser’s review, although important, has some limitations. He did not adopt a systematic methodology and therefore did not include the majority of transgender sport policies. Additionally, Reeser only considered the implications of such policies in relation to elite competitive sport and did not consider the experiences of transgender people who engage in sport or sport-related physical activity for leisure or fitness (e.g. gym fitness activities, jogging).

With the intention of addressing the limitations of the previous literature review, this systematic review has two aims. First, to systematically analyse and critically review the available literature regarding transgender people’s experiences in relation to competitive sport (elite and recreational) and sport-related physical activity participation (e.g. jogging, gym fitness activities). Second, to systematically review the available transgender competitive sport policies with regard to their fairness (i.e. competition in the absence of advantage). It is hoped that this systematic review will further enhance the understanding of sport participation and competition amongst transgender people. It may be expected that as more people define themselves as transgender, the issues that transgender people experience in competitive sport and sport-related physical activity will become more pronounced. It is therefore important that those who work to facilitate and promote sport and develop policies for their own sport organisations (e.g. sport medicine specialists, sport policymakers) are informed about the issues that this vulnerable population face. This will allow for a non-discriminatory atmosphere in sport, whilst ensuring a fair system for all participants and competitors (regardless of their gender identity).

## Methods

### Search Strategy

Preferred Reporting Items for Systematic Reviews and Meta-Analyses guidelines were followed to undertake this systematic review [22]. To obtain relevant peer-reviewed articles, an electronic search of literature published between January 1966 and August 2015 was conducted using the following search engines: ScienceDirect, Web of Science, Scopus and PubMed. Within each search engine, the following search terms were entered: gender dysphoria, gender identity disorder, trans people, trans individual, transgender and transsexual. These terms were combined with three terms relating to sport (physical activity, exercise and sport) using the “AND” operator. The reference lists of eligible papers were searched for potentially relevant publications. Sport policies were obtained through a Google search using the above search terms with the addition of “policy” at the end of all sport-related terms.

### Inclusion and Exclusion Criteria

To address the first aim, articles that were selected were concerned with the experiences and issues surrounding physical activity and sport participation for transgender people. This systematic review only considered articles eligible if they were research articles, as opposed to discussion papers. Case studies were also considered eligible, as research articles were limited. Peer-reviewed articles that were written in English only were included. For the second aim, all available national and international policies on competitive sport in transgender people were selected and reviewed.

### Study Selection

**Fig. 1:**
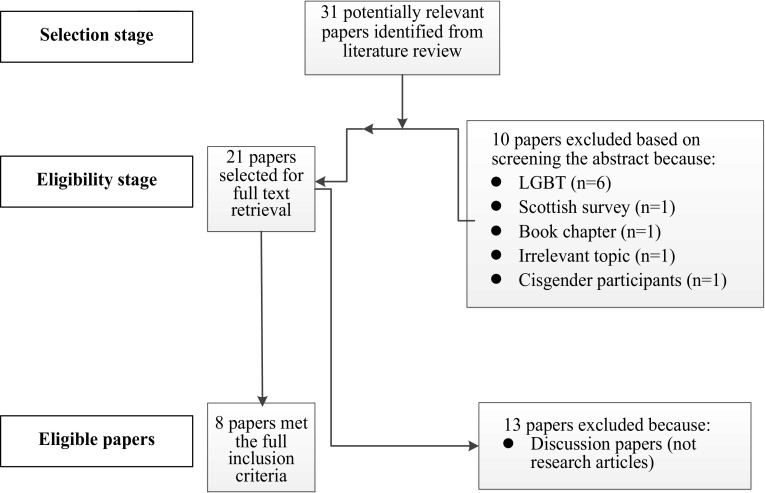
Process of identifying eligible research articles. *LGBT*: lesbian, gay, bisexual, transgender

Thirty-one research articles were considered potentially relevant to the remit of this review. The search also identified 31 competitive sport policies for transgender people. After screening the abstracts, ten research articles were excluded as six were concerned with lesbian, gay, bisexual and transgender sport, one was a Scottish non-academic survey, one was a book chapter, one was concerned with an irrelevant topic and another focused on cisgender participants. The remaining 21 articles were downloaded for full-text review and 13 papers were excluded as they were discussion papers, as opposed to research articles. Therefore, eight research articles fulfilled the inclusion criteria and were consequently included within this systematic review (Fig. 1). All 31 competitive sport policies for transgender people were reviewed and included within this systematic review.

## Results

This section presents the findings from the research articles and sport policies included within this systematic review. First, the findings from the research articles that explored participation in sports (both elite and recreational standards) and sport-related physical activities (i.e. gym fitness activities, jogging) are provided. Second, findings from the reviewed competitive sport policies relating to transgender inclusion are given.

### Transgender People and Sport Participation

#### Characteristics of the Eligible Research Studies

The oldest research article included was published in 2004 [23] and the most recent publication was from 2015 [24]. The majority of the studies were qualitative in nature, all of which employed interviews [24–29]. The remaining two research articles included an experimental study [23] and a cross-sectional survey [30]. Most of the studies were concerned with transgender people who participated in sport competitively, at an elite or recreational level [21, 23, 25–29]. Some authors focused on a specific sport; ice hockey, netball and softball [26, 28, 29] while others were concerned with transgender people engaging in any sport [25, 27, 29]. Broadly, across all sports, Gooren and Bunck [23] explored whether transgender athletes have a physiological advantage in competitive sport. One study explored participation in competitive sports and sport-related physical activity [24] and another study discussed participation in sport-related physical activity only [30]. Details of all of the research articles included within this systematic review can be found in Table 1.

**Table 1:** Study characteristics of research articles included within the review

References	Year	Country	Aim(s)	Sample size (*n*)	Study design	Main finding(s)
Caudwell [25]	2012	UK	To explore two transgender male individuals’ experiences of sport in an educational and recreational environment	2	Qualitative (semi-structured interviews) and a narrative review	Four themes: school sport, their embodied subjectivities, transitioning and sport participation
Cohen and Semerjian [26]	2008	USA	To explore the experience of a transgender female participating in women’s national ice hockey tournaments	1	Qualitative (four open-ended interviews)	Five main themes: policed identity, internal conflict, taint of masculinity, affirmation and creating gender norms
Gooren and Bunck [23]	2004	Netherlands	To explore androgen deprivation and androgen administration in transgender people	36	Retrospective	Androgen deprivation in transgender female individuals increases the overlap in muscle mass with women but does not reverse it
Hargie et al. [24]	2015	UK	To explore transgender people’s experiences of sport in relation to social exclusion and minority stress theory	10	Qualitative (semi-structured interviews)	Four themes: intimidating nature of locker rooms, the impact of alienating sport experiences at school, fear of public space, and being denied the social, health and well-being aspects of sport
Muchicko et al. [30]	2014	USA	To explore the relationship between gender identity and physical activity	80	Cross-sectional survey	Transgender people reported less physical activity and reported lower social support and physical self-perception than the cisgender participants
Semerjian and Cohen [27]	2006	USA	To explore the experiences of transgender athletes, paying particular attention to whether gender identity or performance was related to participation	4	Qualitative (semi-structured interviews)	Athletes discussed a number of barriers and challenges in relation to their sport participation (i.e. incorrect pronoun use, discomfort in changing rooms)
Tagg [28]	2012	Australia and New Zealand	To understand the issues surrounding transgender athletes' sport participation, specifically in relation to men’s netball in New Zealand	2	Qualitative (semi-structured interview) and a narrative review	Transgender-inclusive policies have medicalised gender. Instead of being protective they have done little to make sport fair for transgender participators. Transgender people who are undergoing physical transition have no place to openly participate in netball in New Zealand
Travers and Deri [29]	2011	Canada	To examine the re-negotiation of sex-based boundaries within the context of transgender inclusion in North American lesbian softball leagues	12	Qualitative (semi-structured interviews)	Positive experiences were more often reported by transgender women than transgender men in relation to inclusion despite the re-negotiation of sex boundaries. Several participants perceived testosterone as an athletic advantage in transgender male individuals (when injected) and female individuals (endogenous)

#### Review of Transgender People and Competitive Sport Participation (Elite and Recreational): Research Articles

The same data were extracted from all research articles reviewed (Table 1). Below, we provide the most prominent findings in relation to competitive sport participation from each of these articles. Six research articles were concerned with competitive sport participation within this systematic review [23, 25–29]. The only experimental study was by Gooren and Bunck [23] who aimed to explore whether transgender people taking cross-sex hormone treatment can fairly compete in sport. The authors measured transgender people’s muscle mass (via magnetic resonance imaging) and hormone levels (via urine and blood analyses) before and 1 year after cross-sex hormone treatment. They found that 1 year after transgender male individuals had been administered cross-sex hormone treatment, testosterone levels significantly increased and these levels were within a cisgender male range. They also found that 1 year after cross-sex hormone treatment, transgender male individuals’ muscle mass had increased and was within the same range as transgender female individuals (assigned male at birth) who had not been prescribed cross-sex hormone treatment. In relation to transgender female individuals, Gooren and Bunck found testosterone levels had significantly reduced to castration levels after 1 year of cross-sex hormone treatment. Muscle mass had also reduced after 1 year of cross-sex hormone treatment. However, muscle mass remained significantly greater than in transgender male individuals (assigned female at birth) who had not been prescribed cross-sex hormone treatment.

Therefore, Gooren and Bunck concluded that transgender male individuals are likely to be able to compete without an athletic advantage 1-year post-cross-sex hormone treatment. To a certain extent this also applies to transgender female individuals; however, there still remains a level of uncertainty owing to a large muscle mass 1-year post-cross-sex hormones. While this study was the first to explore, experimentally, whether transgender people can compete fairly, the sample size was relatively small (*n* = 36). Additionally, they did not explore the role of testosterone blockers and did not directly measure the effect cross-sex hormones had on athletic performance (e.g. running time). Many, but not all, transgender female individuals are prescribed testosterone blockers to help them to reach cisgender female testosterone levels, when administration of oestrogen alone is not enough to reduce testosterone levels. This is particularly important if the person aims to undergo gender-confirming surgery, as 6 months of testosterone suppression is a requirement for such procedures. However, if a transgender woman does not wish to undergo surgery or does not wish to have their testosterone blocked to cisgender female levels (e.g. as they wish to use their penis), their testosterone levels will be above cisgender female levels. Differentiating not only between those taking cross-sex hormones and not taking cross-sex hormones, but also transgender female individuals taking testosterone blockers, may be necessary when discussing an athletic advantage.

The remaining studies considered within this section are qualitative, and although they have provided insight into the experiences of transgender people participating in competitive sport, the findings cannot be generalised. Semerjian and Cohen’s [27] narrative account provides a good overview of how diverse and individual the issues and experiences of transgender people participating in competitive sport can be. Some participants felt anxious when engaging in sport because they felt their genitals may be revealed (e.g. when changing). In contrast, one participant used sport as a safe space to escape from the harassment he received at school. It must be considered though, that participants within the study engaged in different sports and their experiences could therefore be associated with the specific sport (i.e. some sports could be more inclusive then others).

Three qualitative studies described the implications that sport policies had on the experiences of transgender people who engaged in sport [26, 28, 29]. Cohen and Semerjian [26] published a case study about a transgender woman (pre-gender-confirming surgery) who was playing in the women’s national ice hockey tournament, but who was eventually banned from playing in the tournament because it was felt she had an athletic advantage. She described how she felt under constant surveillance when she was playing and at times felt ambivalent about what gendered team she should play on. It was apparent that although teammates were supportive, the issues she experienced in relation to inclusion in the tournament were primarily related to constraints put in place by competitive sport policies. Similarly, the discussions held by two former New Zealand transgender female netball players in Tagg’s [28] study gave the impression that although transgender sport policies were supposedly implemented to increase the inclusivity of transgender people, this was not always the case. They discussed how policy would allow a pre-gender-confirming surgery transgender woman to compete in a male or mixed-gender netball team only and they must obey male dress codes. However, the participants in this study were former netball players and therefore their discussions may not have been based on the current state of netball in relation to transgender participation. In contrast to the previously mentioned studies, the majority of participants (*n* = 12) in Travers and Deri’s [29] study discussed the positive experiences they had in relation to transgender participation in competitive sport. However, some of the transgender men did discuss how they had hostile experiences (e.g. incorrect pronoun use). Several of the participants in this study also felt that testosterone gave transgender women (endogenous) and men (when injected) an athletic advantage.

For the two young transgender male individuals in Caudwell’s [25] study, the stage of transition appeared to be instrumental in disengagement from participation in competitive sport. The discussion held by the participants highlighted how accessing sport during their transitional period was difficult as they would not be accepted or feel comfortable on either a male or female team during this period. However, this study again discussed sport very broadly and therefore it is unknown whether the participants’ experiences were associated with specific sports or whether they are generalisable across other sports.

In summary, there is limited research from which to draw any conclusion about whether transgender people have an athletic advantage in competitive sport or not. The limited physiological research conducted to date has informed the development of transgender sport policies that are implemented by sporting organisations all over the world. It is these sport policies that appear to be instrumental in transgender people’s experiences with competitive sport, most of which are negative.

#### Review of Transgender People and Sport-Related Physical Activities: Research Articles

Within this systematic review, only two studies explored sport-related physical activities [24, 30]. Muchicko et al. [30] set out to quantitatively explore the relationship between gender identity and physical activity. They compared levels of physical activity between cisgender and transgender people. The study found that self-identified transgender participants (*n* = 33) reported engaging in less physical activity than cisgender participants (*n* = 47). Social support and self-perception were found to mediate the relationship between gender identity and physical activity. The authors suggested that their study highlights how leisure centres need to be more inclusive, and transgender people need to be given more social support to encourage physical activity. However, this study was limited by the sampling methods employed. The cisgender participants were recruited from a university campus where they potentially had more opportunity to walk around campus, and opportunity for discounted gym memberships, whereas the transgender participants were recruited from a support group for transgender people and were not associated with the university.

As with transgender people who engage in sport at a competitive level, transgender people who engage in sport-related physical activity also appear to experience a range of different barriers. Hargie et al. [24] found in their qualitative study that transgender people prefer to engage in individual, as opposed to group, sport-related physical activities. This was reportedly owing to their fear of being ‘outed’. Regardless of whether sport-related physical activities are engaged in individually or in a group, changing rooms appeared to be a significant barrier. Being excluded from sport-related physical activities was distressing for participants, as they could not maintain physical fitness, which they felt was important in preparation for gender-confirming surgery. Despite these interesting findings, the study is limited by the lack of sociodemographic information provided about participants. Within qualitative research, because of the small sample size, it is often desirable to provide a large amount of sociodemographic detail about participants so that the findings can be interpreted in relation to this information. For instance, in the context of sport-related physical activities, the stage of transition may be an important factor when interpreting the individuals’ current experiences of sport-related physical activities.

The limited research studies concerned with sport-related physical activities suggest that inclusive environments are not created for transgender people engaging in such activities, which may deter engagement.

### Transgender-Inclusive Sport Policies

#### Characteristics of the Eligible Sport Policies

Of the 31 transgender inclusive policies reviewed, 13 were from the USA [31–43]. Ten of the policies reviewed were from the UK [44–53]. One policy was from Australia [54]. The rest of the policies (*n* = 7) were international [18, 20, 55–59]. Details of all of the sport policies included within this review can be found in Table 2.

**Table 2:** Transgender-inclusive sport policies included within this systematic review

Organisation	Pre-puberty	Post-puberty
IOC (2004) [18]	If had GCS, then may complete in line with gender identity	Provide legal recognition of their gender Had GCS Been on CHT for at least 2 years Lived in their newly assigned gender for at least 2 years
IOC (2016) [20]	Transgender male individuals: no restrictions Transgender female individuals: declared gender as female for at least 4 years and have testosterone levels below 10 nmol/L for at least 12 months prior to competition	Transgender male individuals: no restrictions Transgender female individuals: declared gender as female for at least 4 years and have testosterone levels below 10 nmol/L for at least 12 months prior to competition
Amateur Swimming Association (UK; 2015) [48]	IOC 2004 policy is adopted	IOC 2004 policy is adopted
Association of Boxing Commissions (2012) [31]	Allowed to complete in line with gender identity providing they have had GCS	Transsexual female individuals must comply with the IOC Transsexual male individuals must provide legal evidence of their gender and be prescribed CHT Transgender female individuals taking a testosterone suppressant must compete as a male individual until 2 years of medical treatment has been prescribed Transgender male individuals must be being prescribed CHT
Badminton England (UK; 2013) [46]	IOC 2004 policy is adopted	IOC 2004 policy is adopted
British Rowing (UK; 2013) [50]	If hormone treatment has not been started, a transgender female individual may compete as a male individual A transgender girl pre-puberty may compete as a girl or in mixed competition	A transgender male individual may compete as a male individual or in mixed competition Transgender female individuals may compete as female individuals or in mixed competitions providing testosterone levels are within the normal range for a female individual or they have had a gonadectomy If a transgender female individual has not started treatment then they may compete as a male individual or in mixed competition
British Universities and Colleges Sport (UK; 2012) [47]	Not applicable	Recommended that when transgender issues arise, then the policy of each national governing body for that sport should be adopted
Disability Sport Australia (2014) [54]	Encourages participation in line with experienced gender but suggests completion of a TUE form if necessary	Encourages participation in line with experienced gender but suggests completion of a TUE form if necessary
Fédération Internationale de Volleyball (2014) [58]	Gender must be confirmed via birth certificate Female players may be required to submit a gender certificate and/or medical examination	Gender must be confirmed via birth certificate Female players may be required to submit a gender certificate and/or undergo a medical examination
International Tennis Federation (n.d.) [57]	IOC 2004 policy is adopted	IOC 2004 policy is adopted
International Quidditch Association (2015) [59]	Allows players to self-identify	Allows players to self-identify
International Gay and Lesbian Football Association (2014) [56]	Provide legal recognition of their gender Undergo uninterrupted hormone treatment for at least 1 year prior to competition	Provide legal recognition of their gender Undergo uninterrupted hormone treatment for at least 1 year prior to competition
International Association of Athletics Federations (2011) [55]	Endocrine assessment Evidence of GCS Details of post-surgery treatment and monitoring to date	Endocrine assessment Evidence of GCS Details of post-surgery treatment and monitoring to date
Ladies Professional Golf Association (USA; 2010) [43]	A transgender female individual may compete as a female individual if they have undergone GCS Or, a transgender female individual who is treated with testosterone suppression must compete as a man until they have completed hormone treatment for 1 year. After this time they may compete as a woman A transgender male individual who is treated with testosterone may compete in a men’s event but not in a women’s event	A transgender female individual may compete as a female if they have undergone GCS Or, a transgender female who is treated with testosterone suppression must compete as a man until they have completed hormone treatment for 1 year. After this time they may compete as a woman. A transgender male individual who is treated with testosterone may compete in a men’s event but not in a women’s event
Lawn Tennis Association (UK; n.d.) [45]	Allowed to play in line with gender identity providing they have undergone GCS	Surgical anatomical changes have been completed, including external genitalia changes and gonadectomy (removal of ovaries or testes). Legal recognition of their assigned sex has been conferred by the appropriate official authorities CHT has been administered for a sufficient length of time to minimise gender-related advantages in sport competitions Eligibility should begin no sooner than 2 years after gonadectomy
National Collegiate Athletic Association (2011) [32]	To compete on a men’s team, a transgender male individual must be taking CHT and have a diagnosis of gender dysphoria^a^. They are not allowed to play on a women’s team Transgender female individuals must be taking CHT and have a diagnosis of gender dysphoria. They are not allowed to play on a men’s team until they have completed 1 year of CHT A transgender male individual who is not taking CHT may participate on a women’s or men’s team A transgender female individual who is not taking CHT may not compete on a women’s team	To compete on a men’s team, a transgender male individual must be taking CHT and have a diagnosis of gender dysphoria. They are not allowed to play on a women’s team Transgender female individuals must be taking CHT and have a diagnosis of gender dysphoria. They are not allowed to play on a men’s team until they have completed 1 year of CHT A transgender male individual who is not taking CHT may participate on a women’s or men’s team A transgender female individual who is not taking CHT may not compete on a women’s team
Rugby Football Union (UK; n.d.) [51]	IOC 2004 policy is adopted	IOC 2004 policy is adopted
Scottish Football Association (UK; 2008) [49]	IOC 2004 policy is adopted	IOC 2004 policy is adopted
The Football Association (UK; 2014) [44]	Under the age of 16 years, players may play with boys and girls (no GCS required)	Transgender male individuals: must have hormone results within a cisgender male range Undergone CHT for a sufficient amount of time Legal recognition of gender Transgender female individuals: undergone CHT or gonadectomy (removal of testes) Blood results must be within a cisgender female range Legal recognition of gender
UK Roller Derby Association (2014) [53]	No evidence of gender identity or hormone levels is required to participate Must be living full time as their chosen gender	No evidence of gender identity or hormone levels is required to participate Must be living full time as their chosen gender
US Rowing (2015) [35]	All rowers in men’s events are male and all rowers in women’s events are female Gender is determined by legal recognition of gender	All rowers in men’s events are male and all rowers in women’s events are female Gender is determined by legal recognition of gender
US Soccer Federation (2013) [34]	Transgender people are asked to provide legal or another form of documentation to reflect that the athlete’s gender identity is sincerely held and part of their core identity	Transgender people are asked to provide legal or another form of documentation to reflect that the athlete’s gender identity is sincerely held and part of their core identity
USA Gymnastics (2015) [33]	IOC 2004 policy is adopted	IOC 2004 policy is adopted
USA Senior Softball (2014) [36]	IOC 2004 policy is adopted	IOC 2004 policy is adopted
USA Triathlon (n.d.) [37]	Follows the US Anti-Doping Agency rules regarding the use of testosterone, which is a banned substance requiring a TUE to avoid violating policy	Follows the US Anti-Doping Agency rules regarding the use of testosterone, which is a banned substance requiring a TUE to avoid violating policy
USA Boxing (2013) [38]	IOC 2004 policy is adopted	IOC 2004 policy is adopted
USA Sailing (2013) [39]	IOC 2004 policy is adopted	IOC 2004 policy is adopted
USA Track and Field (2005) [40]	IOC 2004 policy is adopted	IOC 2004 policy is adopted
USA Swimming (2013) [41]	Discrimination against any member or participant on the basis of gender, sexual orientation and gender expression is prohibited	Discrimination against any member or participant on the basis of gender, sexual orientation and gender expression is prohibited
Women’s Flat Track Derby Association (UK; n.d.) [52]	Transgender women are allowed to compete as a woman as long as their hormone levels are within a typical female range Information about healthcare provided must be submitted. Transgender male individuals may not participate	Transgender women are allowed to compete as a woman as long as their hormone levels are within a typical female range Information about healthcare provided must be submitted Transgender male individuals may not participate
World Outgames (USA; 2015) [42]	Transgender people are asked to provide legal or another form of documentation to reflect that the athlete’s gender identity is sincerely held and part of their core identity	Transgender people are asked to provide legal or another form of documentation to reflect that the athlete’s gender identity is sincerely held and part of their core identity

*GCS* gender-confirming surgery, *CHT* cross-sex hormone therapy, *IOC* International Olympic Committee, *TUE* therapeutic use exemption, *n.d.* no date

^a^Gender dysphoria is the diagnostic name included within the *Diagnostic and Statistical Manual for Mental Disorders*, Fifth Edition, for people who experience an incongruence between their gender assigned at birth and gender identity [60]

#### Review of the Sport Policies

Policies within this section were systematically reviewed in relation to their inclusiveness of transgender competitors (i.e. maintaining fairness in the absence of advantage for all competitors). The fairness of the policy requirements was judged against the available physiological research that has explored athletic advantage. The time restrictions associated with each requirement were also reviewed (e.g. cross-sex hormones must have been administered for at least 2 years prior to competition). The requirements from each policy are summarised within Table 2 and the most salient points of these policies are then presented in the section that follows.

In 2004, the IOC [18] announced that transgender people who transition after puberty are permitted to compete in sport in line with their experienced gender identity providing they have had gender-confirming surgery, can provide legal recognition of their gender, have been prescribed cross-sex hormone treatment for at least 2 years and have lived in their experienced gender for the same amount of time [18]. Additionally, transgender people who had undergone gender-confirming surgery pre-puberty are eligible to compete in sport in line with their experienced gender identity [18]. This is an international policy and has been adopted by sport organisations all over the world.

While the 2004 IOC [18] policy has been praised for its efforts to address the inclusion of transgender athletes [61], several flaws have been identified [61]. First, the policy excludes transgender people who choose not to have gender-confirming surgery owing to a lack of genital dysphoria (distress), medical reasons, fears about risk during operations, and/or because of other personal reasons [28, 62, 63]. The 2004 IOC [18] policy also excludes transgender people who are in the process of transitioning. For instance, a transgender athlete may be prescribed cross-sex hormone treatment, but be yet to undergo gender-confirming surgery. The 2004 IOC policy [18] therefore adopts a very narrow definition and excludes a large proportion of transgender people [19]. In addition to this, the policy appears to have been developed with only transgender female individuals in mind, possibly as transgender male individuals are not thought to possess athletic advantages in the majority of sports, and therefore the policy discriminates against transgender male individuals [21]. Moreover, the 2004 IOC [18] policy fails to take into consideration the regional, national and international differences in accessing cross-sex hormone treatment and gender-confirming surgery [18, 63–65]. Within this policy, there also appears a lack of an evidence-based rationale as to why a period of 2 years was chosen as the length of time cross-sex hormone treatment must be administered prior to sport competition and why individual differences in blood hormone levels are not considered [66]. As mentioned previously, the role of testosterone blockers in transgender women is also not considered. Although the rationale for the 2-year time period is not made explicit, it may be related to the fact that this time period was imposed by the IOC in 2004, when banning athletes from competitive sport to discipline them for doping violations. The evidence-based rationale for gender-confirming surgery is also not clear [61]; whether an athlete has a penis or vagina appears irrelevant, as this will not change the physiology of the body or the physiological advantage of the person [63].

Approximately 200 days before the 2016 Rio Olympic Games, the IOC announced changes to their competitive sport policy for transgender people. The new 2016 IOC [20] policy suggests that transgender male athletes are able to compete in a male category without any restrictions. Transgender female athletes may compete in a female category if they have declared their gender as female for at least 4 years and their blood testosterone levels are below 10 nmol/L for at least 12 months prior to competition. However, the latter requirement is a general guideline, and each case will be reviewed individually to determine whether 12 months is a sufficient amount of time to suppress testosterone levels to an appropriate level. If transgender female athletes do not meet these requirements, they will be able to compete in a male category. This is a great improvement in sport policy, which considers gender assigned at birth and individual difference in relation to bloody hormone levels and moves away from the requirement of surgery to compete in their experienced gender category. However, we could not find any evidence to support the requirement for testosterone levels to be below 10 nmol/L for at least 12 months.

Despite its flaws, the 2004 IOC policy [18] has been adopted by several other sport organisations. Within this systematic review, 11 sport organisations adopted the policy outlined by the IOC in 2004 [33, 36, 38–40, 45, 46, 48, 49, 51, 57]. All but one (the International Tennis Federation) of these sport organisation policies are employed at a national level. Not only is it problematic that other sport organisations adopted the 2004 IOC policy, but elements of the 2004 IOC policy concerning children pre-puberty are not applicable to sport organisations in the UK and many other countries. Within the UK (and many other countries), children presenting with gender incongruence cannot undergo gender-confirming surgery before the age of 18 years, by which time puberty has usually started.

Three policies stated that it is only necessary to provide legal recognition of gender and to be prescribed cross-sex hormone treatment for a ‘sufficient amount of time’ (international policy) [56] or so that hormone blood levels are within cisgender female or male ranges (national policy) [44, 52]. Policies from the National Collegiate Athletic Association [32] and British Rowing [50] also state that only cross-sex hormone treatment is required; however, the specifics of this requirement differ for both transgender male and female individuals. With both of these policies, transgender female individuals have to provide more evidence of cross-sex hormone treatment and their blood hormone levels in comparison to transgender male individuals. Similarly, the Association of Boxing Commissions [31] in its national policy has different cross-sex hormone treatment requirements depending on gender assigned at birth and how the athlete identifies themselves (transgender or transsexual). The language used within the Association of Boxing Commissions’ policy [31] may be seen as offensive by some transgender people and the difference between “transsexuals” and “transgender” people remains unclear. Policies held by the Ladies Professional Golf Association (international policy) [43] and the International Association of Athletics Federations [55] differ dramatically in relation to gender and gender-confirming surgery as a requirement. In both cases, it is necessary for transgender female individuals to have undergone this procedure, but not for transgender male individuals. Although some of the requirements of these policies are unreasonable and not evidence based (e.g. gender-confirming surgery), the gender difference in relation to the amount of evidence that is required about their gender change seems acceptable considering that only transgender female individuals (and not transgender male individuals) are currently seen to potentially have an athletic advantage [23].

The more inclusive sport policies reviewed here only required legal or medical recognition or do not ask for any evidence of gender; thus they encourage competition in line with the experienced gender (five were national policies and two were international) [34, 35, 41, 42, 53, 54, 59]. The Fédération Internationale de Volleyball [58] had the most invasive policy considered within this systematic review; they ask players to provide a birth certificate to verify gender. Additionally, female players may be asked to provide a gender certificate or submit themselves to a medical examination if the medical evidence is not sufficient. Both British Universities & Colleges Sport [47] and USA Triathlon [37] do not have their own policies, but suggest the adoption of other policies (i.e. those relevant to the sport in question or guidelines of the US Anti-Doping Agency, respectively).

Currently, the majority of sport policies unfairly exclude transgender people from competitive sport, as the requirements they place on them are not underpinned by evidence-based medicine. Until (and if) there is consistent and direct evidence to demonstrate transgender people have an athletic advantage, it seems unreasonable to exclude them on any basis.

## Discussion

The first aim of this systematic review was to explore the experiences of transgender people in relation to competitive sport participation (elite and recreational) and sport-related physical activity. The majority of the studies within this body of literature are qualitative in nature, which may be at least partly a reflection of the low numbers of transgender people in the general population. It is therefore difficult to draw any definite conclusions because of the lack of quantitative research. By its very nature, the findings from qualitative research cannot be generalised but the findings can be used to form a platform from which generalisations can be made. The research articles reviewed here described a generally negative experience of sport participation and sport-related physical activity for transgender individuals. It was evident from these studies that transgender people are facing barriers when engaging in competitive sport and sport-related physical activity. In relation to sport-related physical activity, lack of accessibility to an inclusive and comfortable environment appeared to be the primary barrier to participation. Charities and support organisations working with transgender people should consider developing campaigns to raise awareness about different gender identities. Leisure centres should also be made more aware of potential gender differences (i.e. via training and greater information provision) and be given advice on how to make such environments more inclusive of transgender people (e.g. gender neutral changing facilities with cubicles). In relation to competitive sport participation, the findings from this systematic review suggest that the requirements that transgender competitive sport policies place on competitors were instrumental in transgender athletes’ negative experiences.

While a distinction needs to be made between the issues and experiences transgender people have with regard to participation in sport and competitive sport, it also needs to be acknowledged that there is an overlap. Transgender male and female individuals have anecdotally discussed that access to sport participation (such as becoming part of the local football team) is restricted as even community and local sport organisations who play at a recreational level implement transgender competitive sport policies.

The second aim was to review the available sport policies regarding the fairness for transgender people in competitive sport (i.e. fairness in the absence of advantage). Owing to overinterpretation and fear of the athletic advantage in transgender athletes, the majority of the policies reviewed were discriminatory against transgender people, especially transgender male individuals (i.e. exclusion in the absence of advantage). Although the updated IOC policy may be perceived as more inclusive then the 2004 version, there are still flaws. The requirement for a transgender female individual to have declared their gender as female for at least 4 years is excessive. In the UK and many other countries, once a transgender person has accessed a transgender health service, it is likely to be less than 4 years before a person legally changes their name, undergoes irreversible treatments and, hence, fully commits to their experienced gender. There appears to be a lack of rationale regarding the 4-year time period for transgender athletes, although this time restriction is consistent with the current disciplinary action for cisgender athletes when a doping incident occurs [67]. The 2016 IOC policy [20] also states that to avoid discrimination against transgender female individuals, they are allowed to complete in a male category if they do not meet the requirements for transgender female athletes. For most transgender female individuals, competing in a male category, when their experienced gender is female, would be distressing and may deter engagement in competitive sport altogether. This particular requirement may be promoting exclusion of transgender female individuals in competitive sport, rather than avoiding discrimination.

Several sport policies, including the recently updated IOC 2016 [20] policy, have based their requirements for transgender competitors on indirect, inconsistent and unambiguous evidence. Physiological research involving cisgender people has shown that testosterone deficiency in young men is associated with a decrease in muscle strength [68] and testosterone injections in cisgender men are associated with an increase in some aspects of muscle strength [69]. However, this research did not determine whether these decreases and increases in muscle mass are within ranges for cisgender female and male individuals and the time required to reach cisgender male or female levels. Elbers et al. [70] expanded on this research by exploring the effects of oestrogen supplements and androgen deprivation on fat distribution and thigh muscle mass (by using magnetic resonance imaging) in 20 transgender female individuals. They found that 12 months after cross-sex hormone treatment, transgender female individuals had a more feminine pattern of adiposity and their thigh muscles had decreased. Other research has found that transgender female athletes who have hormonally and surgically transitioned have reported feeling weaker and their testosterone levels tend to be lower than average compared with cisgender women [19, 71]. However, this research does not tell us anything about the relationship between androgenic hormones and athletic ability.

To date, Harper’s study [72] is the only one to directly explore androgenic hormones and athletic ability. The aim of the study was to explore the long-distance (5–42 km) running times of eight transgender female individuals pre- and post-testosterone suppression. It was found that post-testosterone suppression running times were significantly slower in comparison to pre-testosterone suppression. Harper stated that owing to reductions in testosterone and haemoglobin, transgender female individuals post-transition would have the same endurance capabilities as a cisgender female individual. However, the sample size was very small (*n* = 8) and participants were asked to self-report their race times, which might have been subject to recall or social desirability bias.

On average, men perform better than women in sport; however, no empirical research has identified the specific reason(s) why. Based mainly on indirect research with cisgender people, it is commonly believed that androgenic hormones (specifically high testosterone levels) confer an advantage in competitive sports (i.e. enhance endurance, increase muscle mass) and, while this belief has informed several sporting policies, testosterone may not be the primary, or even a helpful, marker in determining athletic advantage [73]. Karkazis et al. [73] have argued that there is no evidence to suggest that endogenous testosterone levels are predictive of athletic performance (apart from doping), as there is variation in how bodies make and respond to the hormone. Testosterone is only one part of a person’s physiology and there are other important factors (both biological and environmental) that should be considered if fairness (the absence of advantage) is the aim in competitive sport. For instance, having large hands is key for manipulation in some sports (e.g. basketball), but this is not seen as an unfair advantage. Establishing what an athletic advantage is in competitive sport would facilitate inclusion of all athletes (regardless of their gender identity) on the premise of fairness.

The Canadian Centre for Ethics in Sport [74] recently released a document offering guidance to sport organisations on how to develop inclusive competitive sport policies for transgender people. An expert panel maintained the viewpoint that everyone has the right to compete in accordance with their gender identity at a recreational and elite level. Cross-sex hormones and gender-confirming surgeries should not be a requirement at any level of sport. If any sport organisation requires transgender competitors to take cross-sex hormones for a specified time, they will have to provide evidence to support that this is reasonable. The panel suggests that when sporting organisations are concerned about safety, based on the size or strength of competitors, such organisations should develop skill and size categories, such as in wrestling.

The issues and challenges that transgender people experience when engaging in competitive sport and sport-related physical activity will undoubtedly become more prominent as the visibility and prevalence of transgender people become more pronounced. Consequently, health professionals working in sport will need to become more familiar with the specific issues and challenges that a transgender person may experience when engaging in sport. By doing this, these professionals will be able to ensure transgender people can start or continue to engage in sport in a safe and inclusive manner. The most common question of people working within the sport domain will likely be: When it is safe and fair to permit a transgender person to compete in sport in line with their experienced gender? At the current time, this is a difficult issue to address considering that there is a lack of direct and consistent physiological performance-related data with transgender people, which is preventing a consensus from being made as to whether transgender people (especially transgender female individuals) do or do not have an athletic advantage. It may be sensible to suggest that until there are direct and consistent scientific data to suggest that transgender competitors have an advantage, transgender people should be allowed to compete in accordance with their gender identity with no restrictions (e.g. no requirement to have cross-sex hormones, gender-confirming surgery). The athletic advantage transgender female individuals are perceived to have (based on indirect and ambiguous evidence) may be no greater than widely accepted physiological (e.g. large hands) and financial (e.g. training opportunities) advantages that some cisgender people possess in competitive sport. Sport organisations wanting to exclude a transgender person from competing in their experienced gender category would need to demonstrate that the sport is gender affected and that exclusion is necessary for fair and safe competition [74, 75]. At the current time, this would be difficult considering there is no evidence to suggest that androgenic hormone levels consistently confer a competitive advantage [74, 75].

### Limitations of the Area and Directions for Future Research

Within the area of sport, physical activity and transgender individuals, research is limited and mainly qualitative. More quantitative research needs to be conducted to increase the applicability and generalisability of the research findings and so that conclusions about transgender people and sport can be drawn. At a medical level, more physiological research is needed with the transgender population to accurately determine whether transgender people have an advantage in competitive sport or not. Future studies should investigate when a person can be considered physiologically as their experienced gender. This in turn should aid more inclusive (i.e. inclusion in the absence of advantage) sport policies for transgender individuals and a fair system for all. To date, the few studies exploring the experiences of transgender people have mainly been concerned with exploring experiences in relation to competitive sport. This research now needs to be extended to those who participate in sport-related physical activity for leisure and fitness. It is also important to understand transgender people’s experiences in the context of different sports. The barriers to, and facilitators of, football participation, for example, may greatly differ to those experienced when engaging in gymnastics, athletics, swimming or aquatic activities. For the latter four sports, clothing may be revealing and an indication of one’s gender. For example, feeling comfortable in swimwear may be an issue for transgender people, especially when they are in the process of transitioning, as the body is often more exposed than in other sportswear (e.g. a football kit) and swimwear is heavily gendered (i.e. swimming trunks are worn by male individuals and swimming costumes by female individuals). In light of this, it would be interesting to explore the experiences of transgender people who have previously participated, or are currently participating, in aquatic activates, gymnastics and/or athletics.

## Conclusion

Overall, it appears that the majority of transgender people have a negative experience of competitive sport and sport-related physical activities. Accessibility to sport-related physical activity needs to be improved. Within competitive sport, the athletic advantage transgender athletes are perceived to have appears to have been overinterpreted by many sport organisations around the world, which has had a negative effect on the experiences of this population. When the indirect and ambiguous physiological evidence is dissected, it is only transgender female individuals who are perceived to potentially have an advantage as a result of androgenic hormones. Within the literature, it has been questioned as to whether androgenic hormones should be the only marker of athletic advantage or, indeed, if they are even a useful marker of athletic advantage. Given the established mental and physical health benefits of engaging in physical activity and sport [13, 14], the barriers transgender people experience are a significant limitation to the promotion of healthy behaviours in transgender individuals. There are several areas of future research required to significantly improve our knowledge of transgender people’s experiences in sport, inform the development of more inclusive sport policies, and most importantly, enhance the lives of transgender people, both physically and psychosocially.
